# Mirror Adaptation in Sensory-Motor Simultaneity

**DOI:** 10.1371/journal.pone.0028080

**Published:** 2011-12-21

**Authors:** Masataka Watanabe, Shion Shinohara, Shinsuke Shimojo

**Affiliations:** 1 School of Engineering, University of Tokyo, Bunkyo-ku, Tokyo, Japan; 2 Division of Biology, California Institute of Technology, Pasadena, California, United States of America; 3 Computation and Neural Systems, California Institute of Technology, Pasadena, California, United States of America; Royal Holloway, University of London, United Kingdom

## Abstract

**Background:**

When one watches a sports game, one may feel her/his own muscles moving in synchrony with the player's. Such parallels between observed actions of others and one's own has been well supported in the latest progress in neuroscience, and coined “mirror system.” It is likely that due to such phenomena, we are able to learn motor skills just by observing an expert's performance. Yet it is unknown whether such indirect learning occurs only at higher cognitive levels, or also at basic sensorimotor levels where sensorimotor delay is compensated and the timing of sensory feedback is constantly calibrated.

**Methodology/Principal Findings:**

Here, we show that the subject's passive observation of an actor manipulating a computer mouse with delayed auditory feedback led to shifts in subjective simultaneity of self mouse manipulation and auditory stimulus in the observing subjects. Likewise, self adaptation to the delayed feedback modulated the simultaneity judgment of the other subjects manipulating a mouse and an auditory stimulus. Meanwhile, subjective simultaneity of a simple visual disc and the auditory stimulus (flash test) was not affected by observation of an actor nor self-adaptation.

**Conclusions/Significance:**

The lack of shift in the flash test for both conditions indicates that the recalibration transfer is specific to the action domain, and is not due to a general sensory adaptation. This points to the involvement of a system for the temporal monitoring of actions, one that processes both one's own actions and those of others.

## Introduction

The ability to change one's behavior according to the observation of another person's experience has no doubt contributed to adaptive behavior in the natural and social environments throughout the evolution of mankind. It is obvious that we learn from the success and failure of others at the cognitive level, where we follow or avoid the observed action/strategy when put in similar situations. Also at the level of motor skills, imitation learning is an important aspect of our motor behavior and has been studied extensively [1], [2], [3], [4], [5], [6]. What is unknown is whether learning from other's experience also occurs at the level of temporal recalibration of sensorimotor relationship [7].

The precise temporal order/timing of self-motor action and external events are crucial as it signals the causal relationship of the two events [8]. In order to correctly judge the temporal order, neural processing has to be achieved in high temporal resolution, despite the fact that neural delay in sensory processing and motor execution may well be in the order of tens of milliseconds and more [9], [10]. Moreover, the neural delay is prone to changes -e.g., due to retinal response times in different lighting conditions [11] or, on longer time scales, due to limb growth [12]. To compensate for this, a dynamic recalibration system is expected. Recently, psychophysical studies have reported effects of temporal recalibration in subjective timing of action and sensory feedback; when the sensory feedback was artificially delayed, the perceived timing of one's own actions eventually became delayed as well [13], [14]. Interestingly, when the delay is taken away after adaptation, the sense of causality is altered, i.e., the subject perceived that the target of control on the screen moved before the computer mouse used for its manipulation – the effect appeared to come before cause [13].

A more classical example of sensorimotor recalibration can be found in the spatial domain with prism adaptation. Prism adaptation is a phenomenon in which the motor system adapts to new visuospatial coordinates imposed by prisms that displace the visual field. Helmoholtz first described adaptation and after-effect on reaching movements in subjects wearing prism glasses [15]. Later, Held and Hein discovered that self-produced movements are necessary for subjects to adapt, postulating that prism adaptation depends on the interaction between the motor and the visual system [16]. A variety of studies that proceeded suggested that the recalibration between sensory and motor system occurs automatically at early/basic levels to cope with changes in the environment.

Though, in many cases where sensory and motor relationships are subject to recalibration, either spatial or temporal, it has been assumed that adaptation occurs only when perturbation in sensory feedback is introduced in one's own action (but see [1]). In this study, we ask if sensorimotor temporal adaptation transfers between individuals. That is, whether simply watching others adjust to delay in auditory feedback (visual-auditory adaptation) leads to shifts in simultaneity judgments of self motion and sound (motor-auditory test), and whether *self adaptation* (motor-auditory adaptation) leads to change in simultaneity judgments of other's motion and sound (visual-auditory test). We have switched the modality between self/other adaptation and other/self test to avoid interference from multisensory adaptation effects. If one's limb was visible during self adaptation and self test, viewpoint invariant (one's own arm and other's arm) “multi-sensory” adaptation between visual limb motion and auditory feedback would mix in to the effect of transfer of “sensorimotor” adaptation between the self and other.

## Results

**Figure 1:**
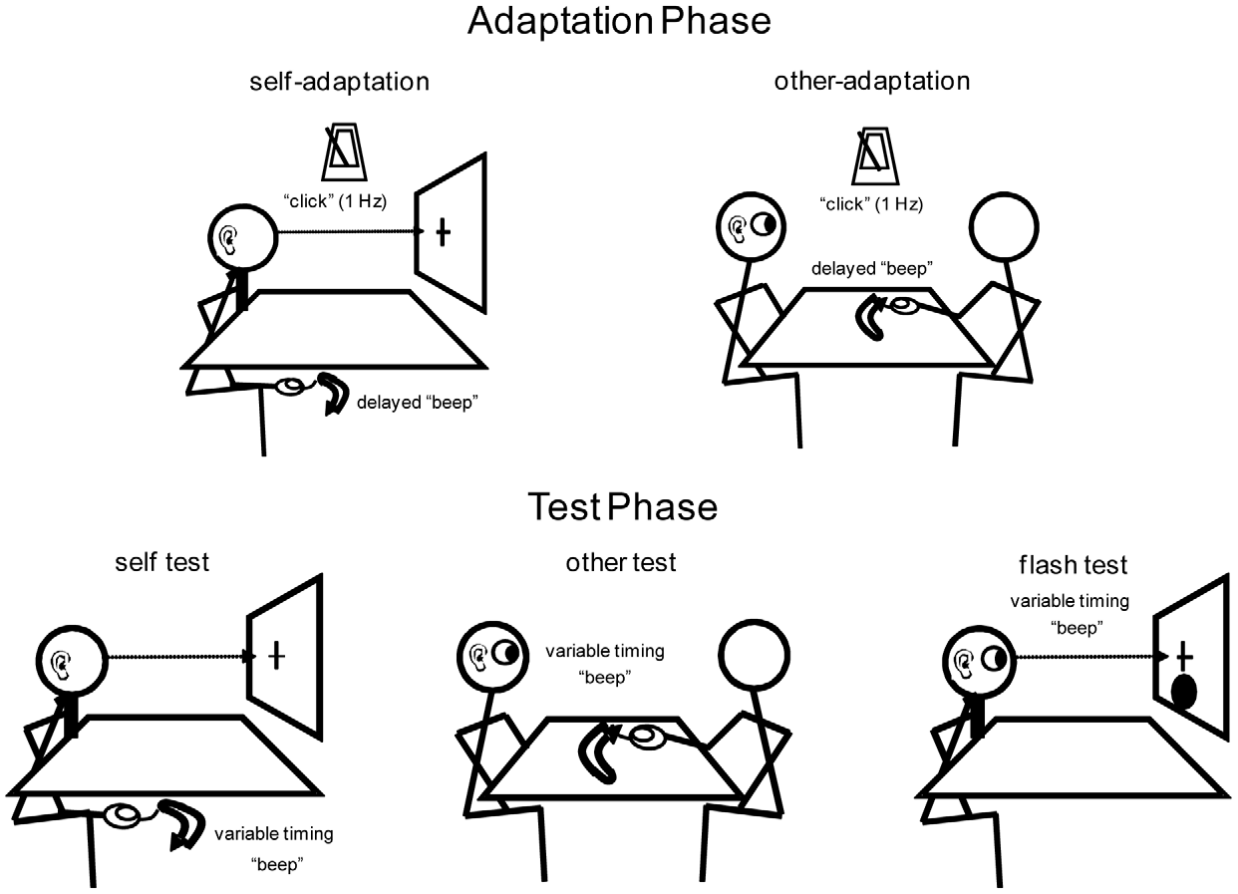
Description of the adaptation phase and the test phase. The type of adaptation depended on the experiment the subject participated in: *other-adaptation* or *self-adaptation* experiment. The *self-adaptation* paradigm was designed to temporally recalibrate one's own action and the auditory feedback. The chin rest, table and point of fixation were set so that the subject could not view his own motor action. Meanwhile, the *other-adaptation* induced temporal recalibration between observation of *other's* action and auditory feedback. A test phase consisted of three types of tests, *self*, *other* and *flash*. The *self test* was designed to measure the point of subjective simultaneity (PoSS) between self motor action and auditory feedback, while the *other test* measured the PoSS between visual observation of other's motor action and auditory feedback. The *flash test* was a control condition to assess PoSS between vision (visual disc) and auditory (beep) events.

Two experiments were carried out, *self adaptation* and *other adaptation* experiment, which shared the general experimental design but differed in who experienced sensorimotor temporal adaptation, self or the other (fig. 1). In both experiments, subjects underwent the following five phases, 1) pre-adaptation test 2) primary adaptation 3) peri-adaptation test 4) secondary adaptation and 5) post-adaptation test. Next, we describe the adaptation phase and the test phase, respectively.

In the adaptation phase, subjects were exposed to a gradually increasing temporal delay in sensory feedback starting from 100 ms to the maximum value of 235 ms while passively observing (*other adaptation* experiment) or performing a motor action (*self adaptation* experiment). Previous studies have used a similar method to gradually increase the temporal delay resulting in robust adaptation [17]. Together, the effect of delay adaptation has been shown to build up in time, even increasing marginally after the removal of delayed feedback [18].

The motor action consisted of a single back-and-forth movement of a computer mouse, and sensory feedback was given at various delays by an auditory beep delivered at the termination of the mouse movement (fig. 1). In the *other adaptation* experiment, subjects passively observed an experimenter manipulate the mouse, whereas in the *self adaptation* experiment, the subject themselves manipulated the mouse in conditions where arm and shoulder were kept out of sight from the subject (see Experimental Procedures). It is important to note that the adapted pair of modalities is different between the two adaptation conditions: sensor-sensory (auditory and vision) for the *other adaptation* and sensory-motor (auditory and motor) for the *self adaptation* experiment.

In both adaptation experiments, a metronome clicking at 1 Hz were used to provide a cognitive temporal target to the subject (in case of the *self adaptation* experiment) or to the experimenter (in case of the *other adaptation* experiment) so as to align the auditory beep delivered at the termination of the mouse movement. In both cases, due to the gradual slow increase of temporal delay, temporal adaptation of motor initiation compensated for the introduced artificial delay so that the metronome and the auditory feedback beep was physically aligned for majority of the time during the adaptation phase. Hence, subjective perceptual temporal recalibration, if any, would occur between auditory-beep/metronome-click and mouse movement termination (visual:*other adaptation* experiment, motor:*self adaptation* experiment).

In the test phase, simultaneity judgments were made in three conditions, i.e. *other*, *self* and *flash*, to assess the point of subjective simultaneity (PoSS) at three levels of adaptation: pre-, peri- and post-adaptation. During the *other* test, the subject observed an experimenter manipulate a computer mouse with sensory feedback (see Experimental Procedures). The motor action was identical to that of the adaptation phase, while the timing of the auditory beep was experimentally manipulated to arrive randomly before and after the termination of the mouse movement. Subjects were required to make two alternative forced-choice subjective simultaneity judgments between the termination of the mouse movement and the auditory beep. Meanwhile, *self* test was identical to the *other* test except that the subjects themselves manipulated the mouse, with their arms and shoulders hidden from view. Finally, during the *flash* test, the subjects made simultaneity judgments between a short duration visual disc (16.7 ms) displayed on a computer screen and the auditory beep (see Experimental Procedures). This is a control condition meant to isolate the effect of audio-visual sensory adaptation. For example, in the *other adaptation* experiment, if transfer of adaptation to both *self* test and *flash* test is observed, it would indicate that both the sensorimotor and the audiovisual system were recalibrated. On the other hand, if transfer of adaptation is limited to the *self* test, it would indicate that audiovisual adaptation between other's motion and auditory feedback led only to sensorimotor adaptation.

**Figure 2:**
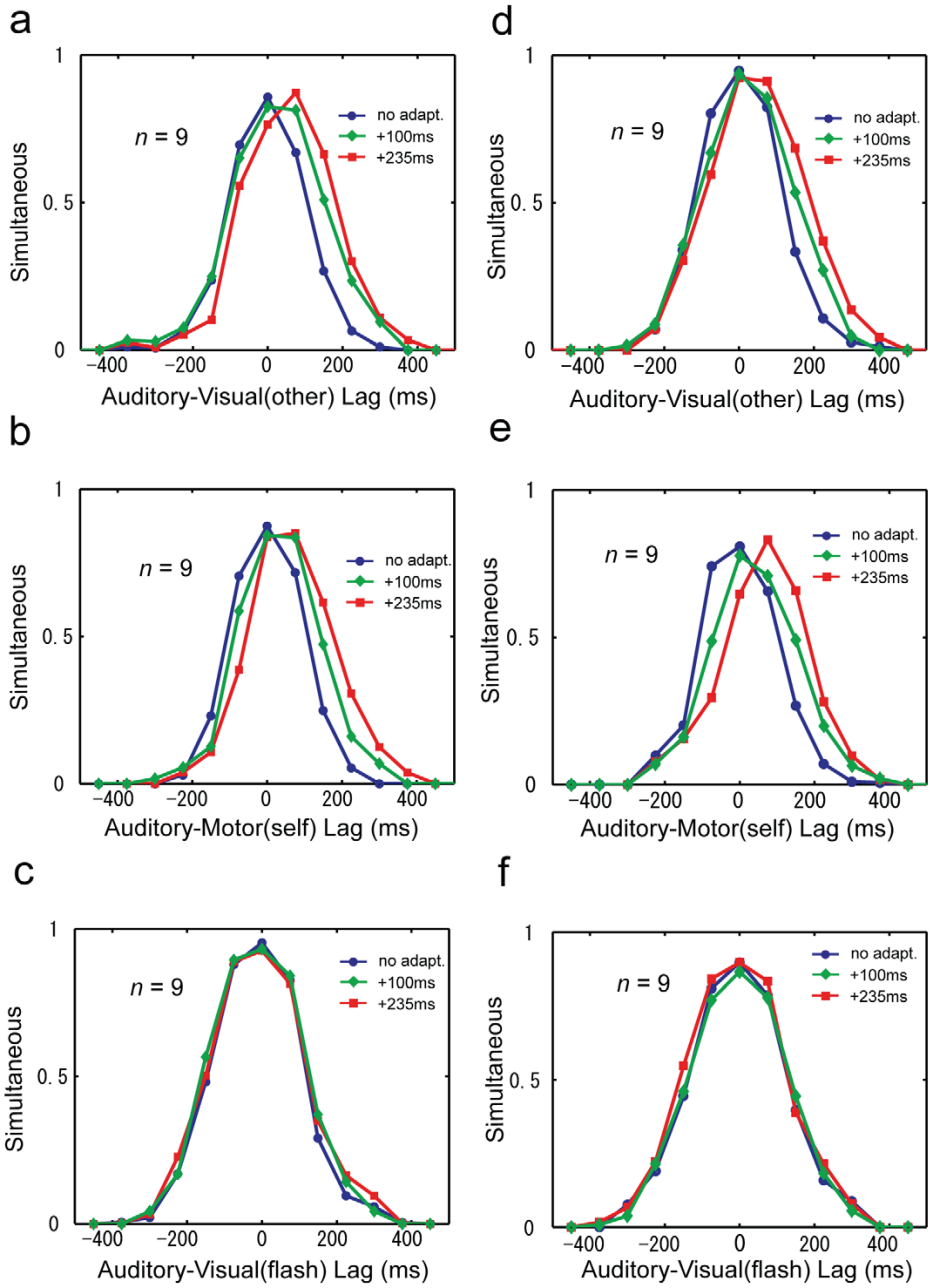
Results of other-adaptation and self-adaptation experiment. The subject averaged results of subjective simultaneity are given for (a) *other adaptation*-*other* test, (b)*other adaptation*-*self* test (c) *other adaptation*-*flash* test (d) *self adaptation*-*other* test, (e) *self adaptation*-*self* test (f) *self adaptation*-*flash* test. The three colored lines denote results of three distinct test phases, pre-adaptation, mid-adaptation and post-adaptation.

Figure 2 shows the result of simultaneity judgments of the two experiments. The vertical columns denote the type of experiment (*self* and *other adaptation*) while the horizontal rows denote the type of test (*self*-, *other*- and *flash* test). The three colored lines in each figure represent the baseline-corrected subject averaged probability of simultaneity judgments obtained from the three test phases, pre-adaptation, peri-adaptation and post-adaptation (see Experimental Procedures). First, we found a basic adaptation effect, where the lag of peak of simultaneity judgment in the test phase was shifted for the adapted condition (Fig. 2a and 2e). Second, we saw a transfer of adaptation from *self* to *other* (Fig. 2b) and *other* to *self* (Fig. 2d). Finally, there was no shift in the *flash* test, in both of the adaptation experiments.

**Figure 3:**
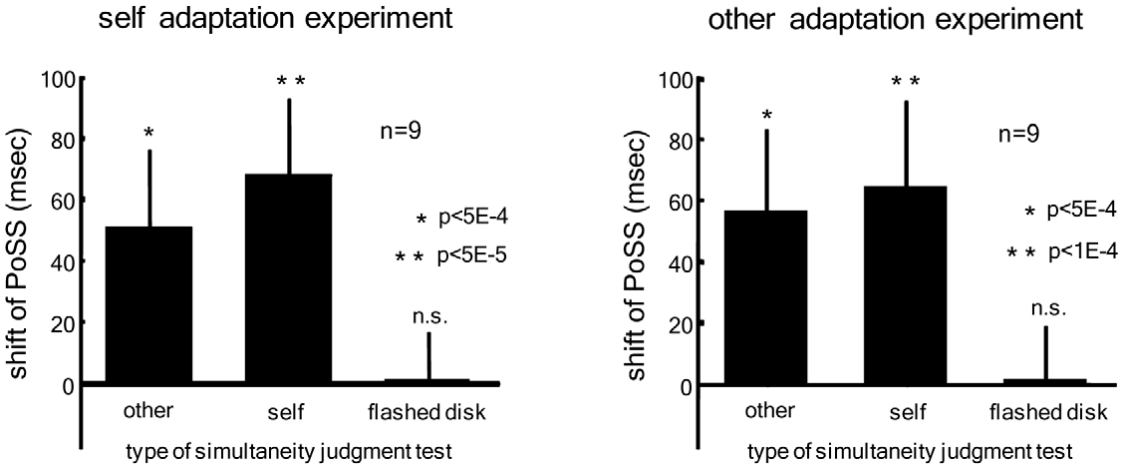
Subject averaged probability of ‘simultaneous’ response as a function of the test delay in the *self/other adaptation* experiment. Statistical analysis indicated that the shifts of PoSS towards the direction of the auditory lag were significant for the *other* and *self* test in both adaptation conditions while the results of *flash* test were not significant for the two adaptation conditions.

To quantify the above results and test for statistical significance, we estimated the PoSS value for individual subjects and test types for the post-adaptation test phase (see Experimental Procedures). Figure 3 shows the difference of PoSS between the pre-adaptation and post-adaptation test phase. Regardless of whether subjects underwent *self adaptation* or *other adaptation*, their results showed significant shifts in the PoSS in the direction to compensate the delay during the adapting phase, thus yielding a negative aftereffect during the testing (Fig. 3a and 3b). No significant shift was found in the *flash* test controls.

Next, we conducted two additional control experiments to test whether the transfer of adaptation between self and other is specific to the action domain. An alternative interpretation of the above results could be object category specific adaptation, say, object specific properties of the computer mouse-auditory feedback were learnt regardless of the actor. In order to test this possibility, we introduced a visual stimulus of an automatically moving mouse and conducted two types of experiments, *automatic mouse adaptation* and *self adaptation with automatic mouse test*. Similar to the movement of the human manipulated mouse in the above experiments, a photograph of a computer mouse moved to the left, then to the right and stopped. Auditory beeps were delivered around the time of mouse stoppage for adaptation and test.

In the *automatic mouse adaptation* experiment, subjects underwent an adaptation procedure basically identical to the *other adaptation* experiment, except that, instead of the experimenter manipulating the mouse, the above mentioned mouse animation was used (see Experimental Procedures). Subjects were exposed to a gradually increasing temporal delay in auditory feedback starting from 100 ms to the maximum value of 235 ms. Two test phases were inserted after delay adaption to 100 ms and 235 ms. A test phase consisted of an *automatic mouse* test and a *self* test. During the *automatic mouse* test, auditory beep was played at a random timing around the time of mouse stoppage and the subjects were required to make a simultaneity judgment of the two events. Meanwhile, the *self* test was identical to the *self/other adaptation* experiments.

**Figure 4:**
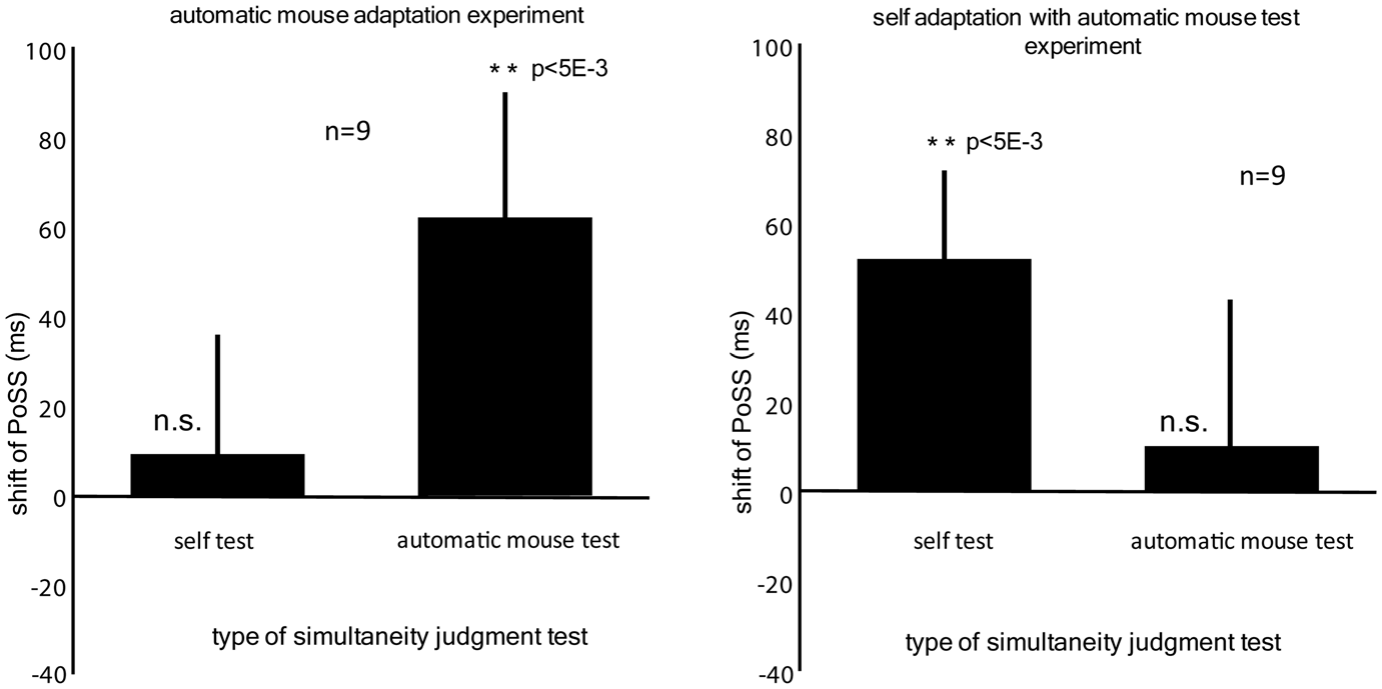
Subject averaged probability of ‘simultaneous’ response as a function of the test delay in the *automatic mouse adaptation* and *self adaptation with automatic mouse test* experiment. Statistical analysis indicated that the shifts of PoSS towards the direction of the auditory lag were significant for the direct adaptation effects (*automatic mouse* test for *automatic mouse adaptation* experiment, *self* test for the *self adaptation with automatic mouse test* experiment), while no statistically significant transfer of adaptation was observed.

In the *self adaptation with automatic mouse test* experiment, subjects were adapted as in the former *self adaptation* experiment. A test phase with two types of tests, *automatic mouse* test and *self* test were inserted after delay adaptation to 100 ms and 235 ms. The results are summarized in figure 4. We find significant shift of PoSS in the direct tests of adaptation (*automatic mouse* test for *automatic mouse* adaptation, *self* test for *self* adaptation), whereas the transfer of adaptation was not observed in both experiments.

## Discussion

The main finding of this study was that sensory motor adaptation transferred inter-personally. Passive observation of other's actions led to a sensorimotor recalibration between the perceived timing of one's own actions and sensory feedback. Likewise, active performance led to recalibration between perceived timing of other's actions and its sensory feedback. This is especially notable given that in the self conditions, the subject could feel their own movements but could not see them; in the other conditions they could see but not feel the movements. Thus the effect shows transfer across sensory modalities [7] as well as across individuals.

The lack of shift in the *flash* test for both experiments indicates that the recalibration is specific to the action domain, and is not due to generalization of crossmodal sensory adaptation. This view is also supported by the additional control experiment, where we found no transfer of adaptation between passive viewing of an automatically moving mouse and self mouse manipulation.

So what is the possible level of recalibration and its possible inputs? Involvement of perceptual learning, as opposed to pure instrumental learning, has been indicated by multiple studies [13], [14] [18], as well as our own. Perceptual learning is defined as the correction of error in the organism's perception of the environment [19]. Hence, as a consequence of perceptual learning, not only the motor behavior but also the perception is modulated. Here, perception points to not only external sensory events but also motor events created by one's own body. Two lines of evidence supporting that sensorimotor delay adaptation involves perceptual learning are, the occurrence of perceptual negative aftereffects (e.g. shifts in temporal order judgements and subjective simultaneity) [13] [14] [18], and the persisting nature of adaptation [18] which is another property of perceptual learning. Next question is where the perceptual learning takes place. One clue comes from the fact that sensorimotor delay adaptation transfers to different type of tasks, i.e. delay adaptation in a visual pacing task altered the perception of an anticipation task [17]. Furthermore, Heron, Hanson and Whitaker have investigated cross-modal transfer effects. Their result show that temporal delay adaptation is robust to the replacement of one modality during the adaptation phase with another modality during the test judgment [7] providing evidence that adaptation takes place beyond the level of modality-specific brain areas. Based on this view, we may hypothesize based on our experimental results that the input to this supra-modal sensorimotor recalibration mechanism is exchangeable between the usual efference-copy/proprioceptive-feedback of self motion and the visual input of other's action. This points to the involvement of a system that processes both one's own actions and those of others. One candidate would be the “mirror system”, a network of neurons that respond similarly to both the performance and observation of actions [20].

In support of the above view, one interesting aspect of our experimental results is the lack of transfer to the *flash* test. Interestingly, robust transfer effects have been reported in a multi-sensory temporal recalibration experiment [21]. Here the transfer of adaptation occurred between stimulus types, i.e. auditory-visual adaptation to temporal shifts between visual disc and auditory beep resulted in the change of percept in the stream/bounce illusion. In terms of modality and stimulus type exchange, the combination of *other* adaptation and the *flash* test has the same configuration, but did not show any transfer effects. This discrepancy can be explained in the following manner. In the case of multi-sensory calibration, since it does not have a motor factor, calibration occurs between two sensory modalities. On the other hand, in sensor-motor delay adaptation, it is evident from cross-modal transfer effects [7] that calibration occurs between motor (efference copy, proprioception) and the readily integrated multi-sensory input. So in theory, during our *other* adaptation phase, there were three possible mechanisms of recalibration 1) audio-visual 2) sensorimotor (motor information from the mirror system) and 3) calibration of both systems. The fact that there was no transfer to the *flash* test and the *automatic mouse* test but to the *self* test indicates that 2) is the only plausible explanation. Meanwhile in the *automatic mouse adaptation* experiment, adaptation did not transfer to the *self* test, suggesting that 1) was the case. Idealistically, the only difference between *other adaptation* and *automatic mouse adaptation* is whether or not there is a human actor manipulating the mouse, providing additional information to the observer in the former case such as intention and kinematics/dynamics of the human movement. Transfer of adaptation to the *self* test in case of the other adaptation, but not in *automatic mouse adaptation*, would provide strong evidence that the mirror system was involved during adaptation. Although, we must admit that our automatic mouse adaptation was not perfect for testing the other alternative explanation, “object category specific adaptation”. We have used a movie of a mouse on the computer screen, instead of a physical mouse actually moving on its own. This lack of “realism” may have played a role in the absence of transfer to the *self* test. Future dedicated studies are needed to confirm the existence or non-existence of yet another interesting recalibration mechanism, “object category specific adaptation”. To summarize, although there are concerns on the validity of the *automatic mouse adaptation* experiment, our set of results suggest that the multi-sensory calibration system and the sensorimotor calibration system are two independent systems, and in our experimental conditions, only one system underwent recalibration for a given adaptation condition.

One interesting point we may note is the counter-intuitive relationship between the perceptual learning aspect of delay adaptation and the observed self-other transfer. Under the assumption that the main role of supra-modal recalibration is to correct for perceptional error arising from limb growth and other bodily changes, it is quite strange that observational information of others was used as a source of recalibration. “Sensorimotor recalibration by observation” fits more nicely to the concept of “world learning”, which involves acquiring new information about the environment and adapting to it [19]. One possible explanation is that the induced change of perception from self-other transfer of delay adaptation is an epiphenomenon arising from neural mechanisms to sustain other functions. Another possibility is that the observed transfer effect does not fit into the current definitions of perceptual learning and world learning; perception modulated by world learning.

In relation to our experimental design, it has been shown that intention plays an important role in the temporal perception of action and sensory feedback. Haggard, Clark and Kalogeras have shown that subjects perceived voluntary movements as occurring later and its sensory feedback as occurring earlier than the physical timing, resulting in shortened perceived difference in timing, while involuntary movements induced by magnetic brain stimulation had reversed effects [8]. In their later study with various control conditions, the temporal attraction in perceived timings were shown to link specifically self initiated actions with their consequences [22]. Together, Waszak *et al.* have reported that temporal attraction effects can also be found in the actual timing of movements, not only in the perceived timing [23]. In our experiment, during self-adaptation, subjects attempted to align an artificially delayed auditory feedback induced by self-motion to an external timing. Although it may not be fully voluntary, the subjects themselves initiated the motion without a direct sensory cue. In the self test, the subjects basically initiated the motion without any goal. Meanwhile, during other-adaptation and other test, there was no aspect of self motor control to begin with, but it is possible that the mirror system conjectured other's intention. We are not in a position to argue the effects of intention due to lack of control in our experiments, but it is a crucial aspect of mirror systems, and an interesting topic for future related studies. Another uncontrolled factor of our experiments is attention. It has been shown that attention has a significant effect on various types of perceptual aftereffects [24], [25], [26]. In our experiment, it is likely that subject's may have directed more attention during self-adaptation compared to other-adaptation, since in the former, subjects were required to align the auditory feedback stimulus to a pacing metronome, while in the latter subjects only passively viewed the experimenter manipulating the mouse. Unfortunately, the experiment was not designed to compare the result of self-adaptation and other-adaptation, say, two different groups of subjects were recruited to avoid effects of residual adaptation from the previous experiment, attention too is an interesting topic for future studies.

Finally, the reported psychophysical effect can be applied to primates and other laboratory animals to investigate an interesting twist regarding the mirror system. The human mirror system is said to have two levels of functions. The first level is that the mirror system serves as the basis of action understanding [27], [28], and the second level is to mediate imitation [29], [30]. The interesting twist is that imitation behavior, which is considered a lower level sub-symbolic ability, cannot be found in monkeys. One account for this is that the human mirror system is qualitatively different from that of other primates, i.e. humans are able to extract low-level kinematic descriptions of movements for imitation [31], [32], [33], [34], [35]. Others have proposed that the human mirror system may encode intention in a manner broadly consistent with other primates, but that it does so in a flexible fashion that enables multiple levels of intentional granularity [36]. We believe that the present findings, “sensor-motor adaptation from observation/modulation of perception of others by self sensorimotor adaptation”, provide an additional tool to investigate the low level function of the mirror system and hence look into the evolution of mirror systems in humans, primates and other species.

## Materials and Methods

### Self-adaptation and Other-adaptation experiment

#### Participants

Ethics approval was obtained from the Institutional Review Board of the University of Tokyo. All of the participants gave written informed consent for their participation. A total of 18 participants (aged between 20 and 26) were used in the experiments; nine participants (six male and three female) in the other-adaptation experiment and nine participants (five male and four female) in the self-adaptation experiment.

#### Stimulus Apparatus

The stimuli were presented using the Psychophysics Toolbox [37], [38] for MATLAB (The MathWorks, Natick, MA) on a Macintosh G4 computer. The visual stimuli for the *flash test* and fixation spot on *self test* and *self-adaptation* appeared on a 21″ CRT monitor with a resolution of 1024 by 768 pixels and a refresh rate of 60 Hz. The viewing distance was 57 cm. A chin rest was used to maintain the participants' head position. Participants used a computer mouse for motor action and a keyboard to make responses.

#### Self and Other Adaptation

The two types of adaptation phases are aimed at recalibrating the subject's sensorimotor timing percept. The type of adaptation, *self* or *other*, was determined by the type of experiment. Half of the 18 subjects participated in the *other-adaptation* experiment and passively viewed an experimenter adapt to gradually increasing temporal delay in sensorimotor feedback. The experimenter and subject sat face to face, seated across a platform (48 cm in height) on which the computer mouse was placed. The motor action consisted of a single back-and-forth movement of a computer mouse and an artificially delayed sensory feedback that was given by an auditory beep delivered after the termination of the mouse movement. The experimenter performed the action repeatedly so as to produce beeps in synchrony with the clicks of a metronome at 1 Hz. The primary-adaptation phase lasted for 10 minutes where the sensorimotor delay was fixed at 100 ms. In the secondary-adaptation phase, the amount of delayed was linearly increased (127, 154, 181, 208, 235 ms), and each of the delay conditions lasted 4 minutes.

The other half of the subjects participated in the *self-adaptation* experiment, and during the adaptation phase, performed the aforementioned motor action themselves. Here the platform was placed under a table (74 cm in height) and the subject took a position assisted with a chin rest and his/her shoulders and arms covered (see Figure 1). The subjects fixated on a fixation spot with diameter of 0.3 degrees which was presented on a computer monitor at a distance of 57 cm from the subject. The subject's task was to temporally align the artificially delayed sensory motor feedback to the metronome running at 1 Hz. The amount of delay and duration of adaptation was identical to the *other*-adaptation phases.

#### Subjective Simultaneity Judgment Tests

Three types of tests, *other*, *self* and *flash*, were performed in a test phase to assess the point of subjective simultaneity (PoSS) in various conditions. First we describe the setup for the three types of test and then explain how they were conducted.

During the *other test*, subjects viewed an experimenter manipulating a computer mouse with auditory sensorimotor feedback (see Figure 1). The setup was identical to that of *other*-adaptation, where experimenter and subject sat face-to-face, seated across a platform (48 cm in height) on which the computer mouse was placed. The motor action was similar to the adaptation phase, consisting of a single back-and-forth movement of a computer mouse, but with intervals of a few seconds between each trial of motion. The auditory beep was delivered before and after the termination of the mouse movement at uniformly random times that spanned ±500 ms. The subject was asked to make a two alternative forced choice (2AFC) on the subjective simultaneity of the termination of mouse movement and auditory beep. During the *self test*, as in the setup of *self-adaptation*, the platform was placed under a table (74 cm in height) and the subject took a position assisted with a chin rest and with his/her shoulders and arms out of view from the subject (see Figure 1). The subjects fixated on a fixation spot with diameter of 0.3 degrees which was presented on a computer monitor at the distance of 57 cm from the subject. They were required to judge the timing between the termination of self-induced mouse motion and the auditory beep.

In the *flash test*, chinrest and monitor setup was identical to that of the flash test. A white visual disc of 8 degrees in diameter appeared below the fixation spot (center eccentricity at 10 degrees), 16.7 ms in duration. The subjects judged the timing of the visual disc and the auditory beep. Metronome was not used during the test phase.

Test trials were coordinated into blocks of 60 trials, where the type of test was fixed within. The type order of test block was randomized and 5 blocks (total of 300 trials) were conducted for each test type during a single test phase. During the peri-adaptation and post-adaptation test phase, top-up adaptation was inserted to maintain the level of adaptation. Three minute worth of adaptation was inserted between every block, where subjects were exposed to the previously described procedure of adaptation, *self* adaptation in the *self adaptation experiment* and *other* adaptation in the *other adaptation experiment*. The temporal delay was fixed at 100 ms for the peri-adaptation test phase and 235 ms for the post-adaptation test phase. The above mentioned blocked procedure of top-up adaptation was used, instead of inserting top-up adaptation between every trial, because the subjects needed to move between setups in particular combinations of adaptation and test (e.g. other adaptation and self test, other adaptation and flash test). Similar blocked top-up adaptation design with a shorter exchange interval was used in a previous study reporting positive effects of crossmodal sensor-sensory temporal recalibration [39]. It is possible that the use of this particular top-up adaptation design lead to attenuation of adaptation effects with short temporal decay, in the order of minutes in our case, and the results are more focused on long-lasting effects of temporal recalibration. The method of constant stimuli was used to quantify three types of subjective simultaneity. For the *self* and *other* test, the actual timing between mouse action termination and auditory stimulus was obtained offline, and subject's responses were binned for further analysis based on this value (more than 20 data points existed in each bin for all subjects). The timing of mouse termination was predicted by the timing of mouse direction reversal and this value was used as reference to decide the randomized onset of the auditory feedback.

#### Data analysis

In order to correct for individual differences in baseline subjective simultaneity judgments, we first estimated the point of subjective simultaneity (PoSS) for the pre-adaptation test phase by fitting a Gaussian function to individual data (probability of ‘simultaneous’ response as a function of the timing difference) with a maximum-likelihood curve fitting method. The estimated pre-adaptation PoSS values from individual subjects and test conditions were independently subtracted from all timing values. Next the timing values were binned together to calculate the average subject response.

### Automatic mouse adaptation and Self adaptation with automatic mouse test experiment

#### Participants

A total of 18 participants (aged between 22 and 29) were used in the experiments; nine participants (five male and four female) in the automatic mouse adaptation experiment and nine participants (three male and six female) in the self adaptation (automatic mouse test) experiment.

#### Stimulus Apparatus

The stimuli were presented using the Psychophysics Toolbox [37], [38] for MATLAB (The MathWorks, Natick, MA) on a Macbook pro computer. Other settings were identical to the above mentioned *self adaptation* and *other adaptation* experiment.

#### Automatic mouse adaptation

Half of the 18 subjects participated in the *self adaptation with automatic mouse test* experiment where the adaptation procedure was identical to the previously mentioned *self adaptation* experiment. Other half participated in the *automatic mouse adaptation* experiment and passively viewed an automatically moving mouse animated on a computer screen. A photograph of a mouse moved to the left (250 ms) and switched its direction to the right (250 ms) and then stopped, which mimicked the mouse motion during adaptation and test in the main experiment. An auditory beep was delivered at a timing relative to the stoppage of mouse motion depending on the stage of adaptation. The next sequence of animation started 500 ms after the stoppage of mouse motion in the previous sequence. The primary-adaptation phase lasted for 10 minutes where the delay was fixed at 100 ms. In the secondary-adaptation phase, the amount of delayed was linearly increased (127, 154, 181, 208, 235 ms), and each of the delay conditions lasted 4 minutes.

#### Subjective Simultaneity Judgment Tests

Two types of tests, self test and automatic-mouse-test, were conducted after adaptation to 100 ms and 235 ms delay. Self test was identical to the one used in the *self/other adaptation* experiment.

In the *automatic mouse* test, subjects viewed an automatically moving mouse animated on a computer screen as in *automatic mouse* adaptation. The auditory beep was delivered before and after the termination of the mouse movement at uniformly random times that spanned ±500 ms. The subject was asked to make a two alternative forced choice (2AFC) on the subjective simultaneity of the termination of mouse movement and auditory beep.

Multiple blocks of 60 trials were performed under the *self* and *automatic-mouse* test. Within a single test phase, the order of test was randomized and a total of 300 trials (5 blocks) were conducted for each test type. The method of constant stimuli was used to quantify two types of subjective simultaneity. For the *self* test, the actual timing between mouse action termination and auditory stimulus was obtained offline, and subject's responses were binned for further analysis based on this value (more than 20 data points existed in each bin for all subjects).
